# A Web-Based Exercise System (e-CuidateChemo) to Counter the Side Effects of Chemotherapy in Patients With Breast Cancer: Randomized Controlled Trial

**DOI:** 10.2196/14418

**Published:** 2019-07-24

**Authors:** Angelica Ariza-Garcia, Mario Lozano-Lozano, Noelia Galiano-Castillo, Paula Postigo-Martin, Manuel Arroyo-Morales, Irene Cantarero-Villanueva

**Affiliations:** 1 Department of Physical Therapy University of Granada Granada Spain; 2 “San Cecilio” University Hospital Granada Spain; 3 Sport and Health University Research Institute University of Granada Granada Spain; 4 Biohealth Research Institute in Granada Granada Spain; 5 Cuídate-Support Unit for Oncology Patients Granada Spain

**Keywords:** breast cancer, chemotherapy, physical fitness, randomized control trial, telehealth, e-health, therapeutic exercise

## Abstract

**Background:**

Breast cancer patients have to face a high-risk state during chemotherapy, which involves deterioration of their health including extensive physical deterioration. Face-to-face physical exercise programs have presented low adherence rates during medical treatment, and telehealth systems could improve these adherence rates.

**Objective:**

This study aimed to evaluate the effectiveness of a Web-based exercise program (e-CuidateChemo) to mitigate the side effects of chemotherapy on the physical being, anthropometric aspects, and body composition.

**Methods:**

A total of 68 patients diagnosed with breast cancer, who were undergoing chemotherapy, were enrolled. The patients were categorized into two groups: e-CuidateChemo (n=34) and controls (n=34). The e-CuidateChemo group participated in an adapted 8-week tailored exercise program through a Web-based system. A blinded, trained researcher assessed functional capacity, strength, anthropometric parameters, and body composition. The intervention effects were tested using analysis of covariance and Cohen *d* tests.

**Results:**

Functional capacity improved significantly in the e-CuidateChemo group compared to the control group (6-minute walk test: 62.07 [SD 130.09] m versus –26.34 [SD 82.21] m; 6-minute walk test % distance predicted: 10.81% [SD 22.69%] m versus –4.60% [SD 14.58%]; between-group effect: *P*=.015 for both). The intervention group also showed significantly improved secondary outcomes such as between-group effects for abdominal (24.93 [SD 26.83] s vs –18.59 [SD 38.69] s), back (12.45 [SD 10.20] kg vs 1.39 [10.72] kg), and lower body (–2.82 [SD 3.75] s vs 1.26 [SD 2.84] s) strength; all *P*<.001 compared to the control group.

**Conclusions:**

This paper showed that a Web-based exercise program was effective in reversing the detriment in functional capacity and strength due to chemotherapy.

**Trial Registration:**

ClinicalTrials.gov NCT02350582; https://clinicaltrials.gov/ct2/show/NCT02350582

## Introduction

A diagnosis of cancer is followed by physical and emotional exhaustion that reduces the quality of life. These functional impairments seem to be aggravated with surgery and radiotherapy plus chemotherapy [1] are linked to a decrease in the level of physical activity of up to 50% [2]. The reduction in physical activity is not only an important deterioration of patients’ physical capacity [3], but also associated with metabolic changes [4], which increase both the recurrence of cancer and the risk of death [5]. During the treatment, a high-risk period occurs wherein patients with breast cancer become especially sensitive; this period involves a deterioration of health, creating a vicious circle that is difficult to break due to the physical and psychological state of the patients.

There has been a growing interest in rehabilitation through physical activity during cancer treatment in the last few years due to the health related-benefits of such rehabilitation. Patients undergoing chemotherapy find it challenging to maintain a physically active lifestyle during their treatment [2], and physical activity programs following the American College of Sports Medicine guidelines [6] are accepted as effective, safe, and well tolerated in patients with breast cancer who are undergoing chemotherapy [7,8]. These programs focus on aerobic, resistance, and stretching exercises with a moderate-high intensity and could successfully address fatigue and quality of life [9] as well as cardiorespiratory fitness, return to work, and body composition [10]. Furthermore, there may be a positive effect of taking part in physical activity programs to optimize chemotherapy completion rates [11]. It is necessary to emphasize the potential clinical implications of this fact, because greater chemotherapy completion rates may improve disease-free and overall survival [12]. Furthermore, exercise could be the key to counter the effects of chemotherapy and radiation during anticancer treatments [13].

Nevertheless, it is not usual for patients with breast cancer to participate in tailored exercise programs during chemotherapy. Several barriers to exercise in these patients, such as time constraints, confusion regarding the safety of returning to exercise, lack of access to standardized breast cancer–specific exercise programs, or cancer- and treatment-related side effects [14], have been identified. The high costs involved in carrying out on-site physical exercise programs is also linked to this situation. A recent study showed that an on-site physical activity program during chemotherapy is not cost-effective for patients with breast cancer, as such an exercise program accounts for 30% of the total costs [15]. Therefore, alternatives to improve these difficulties are urgently needed, with programs that can be adapted according to each patient in terms of intensity and flexibility.

To combat this issue, current technological advances propose a real alternative that has already shown encouraging results. In our recent study, we found that Web-based systems are effective for improving not only the quality of life, pain, muscle strength, and fatigue [16], but also the functional capacity and cognition [17] in survivors of breast cancer. In addition, this program showed a high rate of adherence (93.9%). In fact, new Web-based systems are also effective in producing behavior changes in terms of diet and physical activity [18]. Nevertheless, few studies have addressed this contemporary topic within the chemotherapy field, the majority of which are nonrandomized controlled trials [19-22]. Most previous experiences with telehealth systems for patients undergoing chemotherapy aimed at self-management, patient assessment, coaching, or alerting a clinician [19], but did not seek specific exercise training or the specific intention to avoid worsening of one’s condition. In addition, the literature reveals that self-care systems in patients with chemotherapy are ineffective in managing different symptoms such as fatigue, and therefore, more intervention studies are required to evaluate better strategies for support of cancer patients [23]. A single-arm pilot study [20] proposed a telephone-based exercise intervention to improve fitness, psychological, and anthropometric measures. However, the participants did not receive an adequate tailored intervention [21], or audiovisual material was used [22].

This randomized controlled trial (RCT) aimed to determine the effectiveness of an 8-week low-intensity Web-based therapeutic exercise program for improving the functional capacity, strength, anthropometric parameters, and body composition of patients with breast cancer. We hypothesized that the e-CuidateChemo would prevent the loss of functional capacity and strength and negative changes in anthropometric parameters and body composition after the program in patients with breast cancer undergoing chemotherapy.

## Methods

### Study Design and Participants

This was a two-arm, assessor-blinded, parallel, efficacy RCT (ClinicalTrials.gov: NCT02350582) in which 68 patients with breast cancer undergoing chemotherapy were randomized into the e-CuidateChemo group (n=34) or the control group (n=34). The RCT was performed from September 2013 to June 2015 at a physical therapy laboratory at the University of Granada (Spain). Patients were eligible if they met the following inclusion criteria: diagnosis of stage I-IIIA breast cancer , medical clearance to participate, at the beginning of the chemotherapy, basic ability to use a computer or living with someone who could supervise the first steps using the Web, and having internet access. The participants were excluded if they had a chronic disease or an orthopedic issue that would interfere with the ability to participate in a physical activity program and if they had not provided informed consent.

The Research Ethics Committee of the University of Granada (FIS PI-0457-2010) approved this trial. This trial was performed according to the Helsinki Declaration [24] and the Spanish Biomedical Research Law (14/2007). An oncologist from the chemotherapy unit of the Hospital Virgen de las Nieves (Granada) obtained written informed consent from all participants after the first contact as per the recommendations [25] and recruited the patients according to the established criteria.

### Randomization and Masking

**Figure 1:**
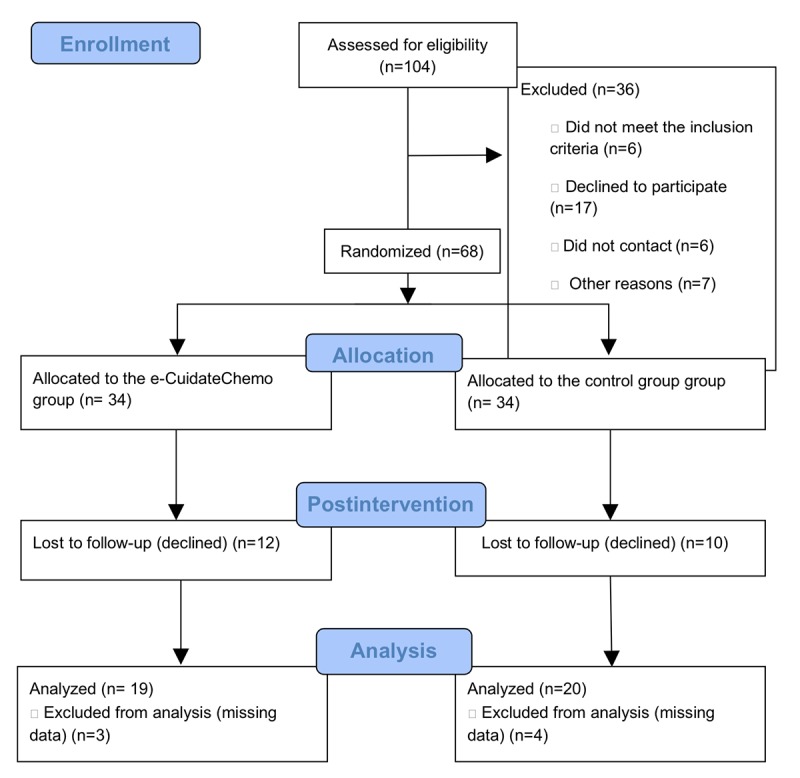
Recruitment and randomization flow diagram process.

After completion of the baseline assessment, the eligible patients were randomized into either the e-CuidateChemo group or the control group by using computer-generated numbers (EPIDAT 3.1, Xunta de Galicia Department of Public Department, Coruna, Spain, and Pan American Health Organization, Washington, DC). The researcher in charge of the assessments, with several years of experience with cancer patients, was blinded to the patients’ randomization (Figure 1). Thereafter, the sequence was introduced by an external member in sealed opaque envelopes that were opened after the baseline assessment.

### Sample Size Calculation

The sample size and power calculations for this trial were obtained through the overall functional capacity using the 6-minute walk test (6MWT). This was considered the principal outcome of this RCT, especially if we take into account the data reported in a previous study that used a similar online rehabilitation system [17]. The sample size was set at 68 participants (34 per group), providing a 90% power (with a 5% significance) and considering a 30% loss to follow-up due to the specific characteristics of this population. The study recruitment was completed when the predefined sample size was reached.

### Intervention

The e-CuidateChemo Intervention is a telerehabilitation program that uses an online system [26] adapted to individual requirements. The system is an 8-week program with three sessions per week (on nonconsecutive days). Each session was organized into a warm up, a main, and a cool down part. The aerobic exercise intensity was between 45% and 60% of the maximum heart rate [27] and lasted for 15-30 minutes. There were a total of 5 strength exercises (Table 1 and Textbox 1) of low intensity with functional implementation. The exercises, that is, their volume and intensity, were adapted for each patient according to the baseline assessment. This online system was previously used in breast cancer survivors and showed very high efficiency [16]. As a result, a specific therapeutic exercise program taking into account the special needs of patients with breast cancer who are undergoing chemotherapy has now been developed.

The e-CuidateChemo system also included a communication system between patients and research staff through an internal service. The research team controlled whether the participants received any additional care apart from our intervention. Moreover, weekly contacts were made to ensure correct performance of the intervention and to adapt the intervention to the participants’ chemotherapy cycles.

The control group received the usual care with some written basic recommendations for physical exercise, following the general recommendations of the American College of Sports Medicine [6]. The research team controlled the changes in the level of physical activity of these participants through the International Physical Activity Questionnaire. Once the intervention was complete, the control participants were offered participation in the same Web-based exercise program as the intervention group for ethical reasons. No data were recorded from these patients.

**Table 1:** Resistance exercises through the e-CuidateChemo telehealth program.

Material and week		Time (min)	Volume	Intensity criteria (Borg rating)
**Self-supporting**				10-13^a^
	1	15	Training: 1×10-12 repetitions	
	2	15	1×12 repetitions	
	3	20	(2×8 repetitions) 30 seconds	
	4	20	(2×10 repetitions) 30 seconds	
**Elastic bands**				10-13^a^
	5	20	Training: 1×12 repetitions	
	6	25	(2×12 repetitions) 30 seconds	
	7	25	(3×8 repetitions) 30 seconds	
	8	20	(2×12 repetitions) 30 seconds	

^a^Two days with more than a rating of 13 on the Borg scale after training represents a decrease in the intensity of the training program.

**Resistance exercises.**

Push up: in standing (wall) or prone position (lying down) with/without support

Squat: lifting arms at 90°

Rowing: in semisquat position

Lunge: front and side

Circular movement of the legs in supine position

### Outcomes

All outcomes were assessed at baseline and after the 8-week program. A clinical and sociodemographic questionnaire was used for the assessments.

#### Principal Variable: Functional Capacity

The 6MWT is a useful measure of functional capacity [28] (H-P-COSMOS for graphics, Germany) [29]. Prior to the test, all patients were familiarized with the treadmill protocol through training performed 2 hours before starting the test and with the period of rest. The participants were instructed to walk as far as possible for 6 minutes [28]. The 6MWT is an objective and reliable test (with an intraclass correlation coefficient [ICC]=0.88) [29]. The results of the 6MWT expressed as percent predicted values were calculated using the reference equation described previously by Enright and colleagues [30]. Finally, participants were distributed for secondary analyses as per their physical activity capacity (normal vs impaired) by using the 75% cutoff, according to Enright and colleagues [30].

#### Outcomes Variables

##### Abdominal Strength

The abdominal endurance was measured with the patient lying on their back and their knees bent. The patients were instructed to keep the following position as long as possible: arms lifted with the palms guided to the level of the knees, avoiding the lower angle of the scapula from rising from the surface. The research team encouraged the patients and registered the number of seconds they held the position (max of 90 seconds). This is a reliable test with an ICC of 0.97 [31].

##### Lower-Body Strength

In the multiple sit-to-stand test, participants were asked to sit down and stand up from a chair 10 times as fast as possible. The research staff recorded the length of completion of the test in seconds, which had a good reliability, with an ICC of 0.80 [32].

##### Lumbar Strength

The lumbar resistance was evaluated using an analog dynamometer (TKK 5002 Back-A, Takey, Tokyo, Japan). The participant was to assume a standing position and maintain a position of 30 degrees. The test was repeated three times, with a 1-minute delay between measurements. Finally, the average of the three measurements was recorded. This test has demonstrated a high reliability, with an ICC of 0.81-0.85 [33].

##### Handgrip Strength

A digital dynamometer (TKK 5101 Grip-D, Takey, Tokyo, Japan) with an adjustable grip was used to measure the upper-body muscular strength, registering the average score for each hand (the test was repeated three times with 1-minute delay between measures). This test is valid and reliable [34].

##### Anthropometric and Body Composition Outcomes

The waist and hip circumferences were measured using a plastic tape measure. To assess the waist circumference, the plastic tape was placed midway between the lower rib margin and the top of the iliac crest. To measure the hip circumference, the plastic tape was placed at the level of the greater trochanter. These measures have demonstrated a high reliability, with ICCs of 0.89 and 0.81 for the waist and hip circumferences, respectively [35].

We used bioelectrical impedance (InBody 720, Biospace, Gateshead, UK) to measure the body composition. The instrument has a high reliability (ICC=0.98) [36].

### Statistical Analysis

A descriptive analysis was performed, and the mean, 95% confidence interval, and SDs were calculated for each group. To check the differences between groups at the baseline, we used the Student *t* test and Chi-square test. We also used the Chi-square test to calculate the changes in physical activity capacity after the intervention. Normal distribution of the variables was proved with the Shapiro-Wilk test.

Analysis was conducted according to the intention-to-treat principle (with the worst value carried forward in patients who had missing data). The intervention effects on study variables were tested using repeated measure ANCOVA. The time since diagnosis, age, stage of breast cancer, type of surgery, and menopausal status were used as covariates. Regarding the level of significance, interaction effects were reported (5% level of significance). If the analysis revealed a significant interaction, we performed pairwise comparisons with the Bonferroni adjustment to determine if there were differences in the scores between groups. Moreover, the effect size was calculated using Cohen *d* values. The Statistical Program for Social Sciences (version 22.0; IBM, SPSS Statistic for Windows, Armonk, NY) was used for statistical analyses.

## Results

### Sociodemographic and Clinical Data

In summary, 68 patients met the inclusion criteria and were randomized into either the e-CuidateChemo group (n=34; mean age 48.82 [SD 7.68]) or the control group (n=34; mean age 47.32 [SD 9.92]). Figure 1 shows the flow chart of patient distribution and the number and reasons for dropouts. There were 12 dropouts (35.29%) in the e-CuidateChemo group and 10 dropouts in the control group (29.4%). Moreover, three participants from the e-CuidateChemo group and four participants from the control group were excluded from the main analysis due to missing data. Adherence rate for the e-CuidateChemo group, calculated as a ratio of the number of exercise sessions performed in relation to the number of sessions prescribed, was 73.33%. The sociodemographic and medical characteristics are shown in Table 2. None of the participants reported receiving any additional support care in addition to the study program.

**Table 2:** Demographic, clinical, and medical characteristics of the e-CuidateChemo and control groups.

Characteristic		e-CuidateChemo group (n=34)	Control group (n=34)	*P* value^a^
Age (years), mean (SD)		48.82 (7.68)	47.32 (9.92)	.59
**Marital status, n (%)**				.51
	Single	4 (18.2)	2 (9.1)	
	Married	16 (72.7)	16 (72.7)	
	Divorced/widowed	2 (9.1)	4 (18.2)	
**Educational level, n (%)**				.81
	Basic	9 (40.9)	8 (36.4)	
	Medium	6 (27.3)	8 (36.4)	
	High	7 (31.8)	6 (27.3)	
**Employment status, n (%)**				.49
	Housewife	3 (13.6)	2 (9.1)	
	Employed	6 (27.3)	4 (18.2)	
	Medical leave/unemployed (by illness)	12 (54.5)	12 (54.5)	
	Unemployed/retired	1 (4.5)	4 (18.2)	
**Tumor stage, n (%)**				.79
	I	7 (31.8)	5 (22.7)	
	II	10 (45.5)	11 (50.0)	
	IIIA	5 (22.7)	6 (27.3)	
**Chemotherapy cycles, n (%)**				.44
	4	5 (22.7)	2 (9.1)	
	5-7	5 (22.7)	7 (31.8)	
	8	12 (54.5)	13 (59.1)	
**Type of surgery, n (%)**				.48
	None	10 (45.5)	8 (36.4)	
	Lumpectomy	3 (13.6)	7 (31.8)	
	Quadrantectomy	5 (22.7)	5 (22.7)	
	Mastectomy	4 (18.2)	2 (9.1)	
**Menopausal status, n (%)**				.22
	Premenopausal	11 (50.0)	15 (68.2)	
	Postmenopausal	11 (50.0)	7 (31.8)	
**International Physical Activity Questionnaire score, n (%)**				.89
	Low (<500 MET^b^-min/week)	6 (28.6)	7 (31.8)	
	Moderate (500-4499 MET-min/week)	11 (52.4)	12 (54.5)	
	High (≥4500 MET-min/week)	4 (19.0)	3 (13.6)	

^a^*P* values for intergroup comparisons using Student *t* test or Chi-square test, as appropriate.

^b^MET: metabolic equivalent of task.

### Effects of the e-CuidateChemo Intervention on Functional Capacity

Significant interaction effects were found for both 6MWT (*F*_1,37_=6.51; *P*=.015) and the percentage of 6MWT prediction (*F*_1,37_=6.44; *P*=.015). We found a significant difference in favor of the intervention group for the walked distance and the 6MWT predicted percentage (both *P*=.015), with distance and percentage increasing in the intervention group but decreasing in the control group. After the intervention, the effect size values were large for both 6MWT (*d*=0.83; 95% CI –32.23 to 33.91) and 6MWT predicted percentage (*d*=0.83; 95% CI –4.96 to 6.63; Table 3). We found no changes in the results after inclusion of the covariates.

**Table 3:** Within-group and between-group effects for physical outcomes at baseline and after the 8-week intervention. Data are shown as mean (SD) and 95% CI for the mean at the baseline and after the 8-week intervention and as the mean difference and 95% CI for the differences for within- and between-group effects.

Parameter			e-CuidateChemo group (n=19)	Control group (n=20)	Between-group effects
**Functional capacity**					
	**6-minute walk test (m)**				
		Baseline	421.38 (176.53); 36.30-506.47	480.13 (134.98); 416.96-543.30	N/A^a^
		8-week intervention	483.46 (149.37); 411.46-555.45	453.79 (99.98); 406.99-500.59	N/A
		Within-group effect - baseline to 8 weeks	62.07 (130.09); –0.63 to 124.77	–26.34 (82.21); –64.81 to 12.13	–88.41; –158.64 to –18.18^b,c^
	**6-minute walk test % predicted (m)**				
		Baseline	74.52 (27.32); 61.35-87.69	85.23 (23.29); 74.32-96.13	N/A
		8-week intervention	85.34 (20.33); 75.54-95.14	80.62 (16.33); 72.98-88.27	N/A
		Within-group effect - baseline to 8 weeks	10.81 (22.69); –0.11 to 21.75	–4.60 (14.58); –11.42 to 2.21	–15.42; –27.73 to –3.11^b,c^
**Strength**					
	**Abdominal strength (s)**				
		Baseline	29.01 (27.29); 15.85-42.16)	48.60 (46.07); 27.04-70.16	N/A
		8-week intervention	53.94 (39.03); 35.13-72.76	30.01 (17.90); 21.63-38.39	N/A
		Within-group effect - baseline to 8 weeks	24.93 (26.83); 12.00-37.87	–18.59 (38.69); –36.69 to –0.48	–43.74; –64.88 to –22.60^c,d^
	**Lower-body strength (s)**				
		Baseline	24.30 (4.53); 22.11-26.49	23.23 (3.54); 21.57-24.89	N/A
		8-week intervention	21.47 (3.58); 19.74-23.20	24.50 (4.32); 22.48-26.52	N/A
		Within-group effect - baseline to 8 weeks	–2.82 (3.75); –4.63 to –1.01	1.26 (2.84); –0.06 to 2.60	4.11; 2.01-6.21^c,d^
	**Back strength (kg)**				
		Baseline	41.05 (15.06); 33.79-48.31	39.27 (15.14); 32.18-46.36	N/A
		8-week intervention	53.50 (16.01); 45.78-61.22	40.66 (13.88); 34.16-47.16	N/A
		Within-group effect - baseline to 8 weeks	12.45 (10.20); 7.53-17.37	1.39 (10.72); –3.62 to 6.41	–10.59; –17.27 to –3.90^b,c^
	**Handgrip strength - affected side (kg)**				
		Baseline	23.41 (6.62); 20.21-26.60	23.76 (3.77); 22.00-25.53	N/A
		8-week intervention	25.45 (5.94); 22.58-28.32	25.08 (4.46); 22.99-27.16	N/A
		Within-group effect - baseline to 8 weeks	2.04 (2.75); 0.71-3.36	1.31 (3.70); –0.42 to 3.04	–0.79; –2.86 to 1.27
	**Handgrip strength - nonaffected side (kg)**				
		Baseline	24.03 (5.01); 21.61-26.44	24.72 (4.42); 22.65-26.79	N/A
		8-week intervention	24.74 (5.00); 22.32-27.15	24.70 (4.33); 22.67-26.73	N/A
		Within-group effect - baseline to 8 weeks	0.71 (1.82); –0.16 to 1.59	–0.01 (2.19); –1.04 to 1.01	–0.78; –2.07 to 0.49

^a^N/A: not applicable.

^b^*P*<.05 (significant between-group effect).

^c^Large effect size: Cohen *d*>0.8.

^d^*P*<.001 (significant between-group effect).

Table 4 shows the differences between patients with a normal physical activity capacity and those with an impaired physical activity capacity after the intervention. There was an increase in the number of participants recovering the normal exercise capacity in the e-CuidateChemo group (45.5% to 78.9%, pre-postintervention) compared to the decrease in the control group (73.3% to 65%). Statistical analysis revealed significant changes between the two groups (*P=*.02).

**Table 4:** Baseline, postintervention, and change in physical exercise capacity. Data are shown as frequencies (percentages) for baseline and postintervention and as frequency differences for change (including loss to follow-up).

Time point	e-CuidateChemo group		Control group	
	Normal physical exercise capacity	Impaired physical exercise capacity	Normal physical exercise capacity	Impaired physical exercise capacity
Baseline, n (%)	10 (45.5)	12 (54.5)	17 (77.3)	5 (22.7)
Postintervention, n (%)	15 (78.9)	4 (21.1)	13 (65.0)	7 (35.0)
Change^a^, n	5	–8	–5	2

^a^Impaired to normal physical exercise capacity.

### Effects of the e-CuidateChemo Intervention on Muscle Strength

The ANCOVA revealed significant interaction effects for abdominal strength (*F*_1,38_=17.55; *P*<.001) and back strength (*F*_1,38_=10.28; *P*=.003). The e-CuidateChemo group showed an increase in abdominal and back strength (both *P*<.001) after the intervention compared with the control group, which led to a decrease in abdominal strength and back strength at the baseline (Table 3). We also obtained a significant interaction effect for lower-body strength (*F*_1,38_=15.74; *P*<.001). In this case, the e-CuidateChemo group showed an improvement in their lower-body strength after the intervention (*P*<.001), while the control group showed similar results at the baseline (Table 3). The intergroup effect size was large for all variables, namely, abdominal strength (*d*=1.33; 95% CI –8.88 to 11.55), lower-body strength (*d*=–1.26; 95% CI –2.27 to –0.251), and back strength (*d*=1.08; 95% CI –2.11 to 4.28). The inclusion of the covariates did not change the results of any of the variables. Regarding the other strength-related measures, handgrip strength for the affected and nonaffected sides did not reveal significant interaction effects (*F*_1,38_=0.60; *P=*.44 and *F*_1,38_=1.54; *P=*.22, respectively).

### Effects of the e-CuidateChemo Intervention on Anthropometric Parameters and Body Composition

The repeated-measure ANCOVA analyses did not show any significant interaction effects for any of the variables, namely, waist and hip circumferences, weight, body fat, lean mass, and body mass index (Table 5).

**Table 5:** Within-group and between-group effects for anthropometric and body composition variables at the baseline and after 8-week intervention. Data are shown as mean (SD) and 95% CI for the mean at baseline and 8-week intervention and as mean differences and 95% CI for the differences for within- and between-group effects.

Parameter		e-CuidateChemo group (n=19)	Control group (n=20)	Between-groups effects
**Waist circumference (cm)**				
	Baseline	85.20 (11.66); 79.81-90.74	86.10 (8.70); 82.02-90.17	N/A^a^
	8-week intervention	86.01 (11.07); 80.83-91.19	86.28 (10.88); 81.19-91.37	N/A
	Within-group effect - baseline to 8 weeks	0.73 (3.41); –0.86 to 2.33	0.18 (3.47); –1.43 to 1.80	–0.55; –2.75 to 1.65
**Hip circumference (cm)**				
	Baseline	101.58 (9.59); 97.08-106.07	103.82 (8.74); 99.72-107.91	N/A
	8-week intervention	102.72 (8.60); 98.69-106.75	103.50 (9.59); 99.01-107.99	N/A
	Within-group effect - baseline to 8 weeks	1.14 (3.20); –0.35 to 2.64	–0.31 (1.88); –1.19 to 0.56	–1.46; –3.14 to 0.22
**Weight (kg)**				
	Baseline	66.46 (12.29); 60.70-72.21	67.82 (10.47); 62.91 to 72.72	N/A
	8-week intervention	67.35 (11.27); 62.07-72.62	68.20 (11.71); 62.72 to 73.68	N/A
	Within-group effect - baseline to 8 weeks	0.89 (2.89); –0.46 to 2.24	0.38 (2.57); –0.82 to 1.59	–0.50; –2.26 to 1.25
**Body fat (%)**				
	Baseline	32.90 (9.60); 28.41-37.39	35.01 (7.32); 31.57-38.43	N/A
	8-week intervention	33.41 (9.01); 29.19-37.63	33.13 (6.69); 29.99-36.26	N/A
	Within-group effect - baseline to 8 weeks	0.51 (2.17); –0.50 to 1.52	–1.87 (7.04); –5.17 to 1.42	–2.38; –5.72 to 0.95
**Lean mass (kg)**				
	Baseline	23.84 (2.61); 22.61-25.06	23.76 (3.15); 22.28-25.23	N/A
	8-week intervention	23.93 (2.75); 22.64-25.22	23.93 (3.32); 22.37-25.48	N/A
	Within-group effect - baseline to 8 weeks	0.09 (1.05); –0.39 to 0.58	0.17 (1.05); –0.32 to 0.66	0.07; –0.59 to 0.74
**Body mass index (kg/m^2^)**				
	Baseline	26.31 (4.97); 23.98-28.63	26.79 (3.79); 25.01-28.56	N/A
	8-week intervention	26.64 (4.58); 24.49-28.78	26.89 (4.32); 24.87-28.91	N/A
	Within-group effect - baseline to 8 weeks	0.33 (1.09); –0.18 to 0.84	0.10 (1.02); –0.37 to 0.58	–0.22; –0.90 to 0.45

^a^N/A: not applicable.

## Discussion

The results of this RCT show that a Web-based exercise program is effective in reversing the detriment in functional capacity and strength, which reflects a physical deterioration normally experienced by patients with breast cancer who are undergoing chemotherapy. Having an adequate physical condition during chemotherapy improves the health state of the patients [37-40], reduces side effects, allows modulations of the response to chemotherapy [41], and can even decrease the size of tumors [42]. Therefore, the findings of this RCT provide evidence about an adequate support for patients with breast cancer during chemotherapy.

This therapeutic program involved an improvement, with significant differences in the walked distance of the 6MWT in the e-CuidateChemo group as compared to the control group. The e-CuidateChemo group had a large effect size, showing its effectiveness despite the low intensities and volumes of aerobic exercises (up to a maximum of 30 min at 60% of the maximum heart rate). This increase experienced by the e-CuidateChemo group in the walked distance is above the smaller change considered clinically relevant in cancer patients (43.1 m) [43,44].

In our previous randomized trial with breast cancer survivors [17], a moderate-intensity Web-based exercise program showed an improvement of 104.84 m after 8 weeks. However, the program involved more specific training tailored to improve cardiorespiratory fitness (the American College of Sports Medicine) and the participants had finished a medical treatment. Other previous randomized studies on face-to-face exercise programs also found improvements with aerobic fitness, but all of them involved more specific and intense aerobic exercise programs [45]. The current evidence seems to indicate a higher gain of physical fitness with moderate- or high-intensity exercise programs [46]. A Web-based support system could be useful for encouraging patients to avoid the barriers of exercise [47].

It is also important to emphasize the presence of a significant difference between groups in terms of the 6MWT percentage predicted, which may also be used to identify the decrease in the exercise capacity [30]. We found an increase of 10.8% in the e-CuidateChemo group (with an average change between 74.5% and 85.3%) and a decrease in the control group (change from 85.2% to 80.6%). Within the e-CuidateChemo group, 33.4% of patients with breast cancer reached a normal exercise capacity. In contrast, 12.3% of participants in the control group reduced their physical exercise capacity after 8 weeks. These results were slightly lower than those reported in more specific previous studies [17,45]; in contrast, the results of van Waart and colleagues [11] confirm that low-level physical exercise can help minimize the decline in cardiorespiratory fitness. This fact is very important. Evidence shows that the improvement of cardiorespiratory fitness could help offset the medical treatment–related side effects such as heart damage [48,49]. In addition, it could modulate the response to chemotherapy [41]. Therefore, these data justify the need to integrate such an intervention in the care routine of patients with breast cancer during their treatments.

The Web-based exercise program also achieved an improvement in almost all estimations of muscle strength. The results showed significant differences in abdominal, lower-body, and back strength, with a difference of 43.74 s, 4.11 s, and 10.59 kg, respectively, between both groups. We also found an improvement of 85.9% (abdominal strength), 11.6% (lower-body strength), and 29.74% (back strength) in the e-CuidateChemo group compared with the control group (deterioration of 38.2% and 5.4% and an improvement of only 3.53%, respectively). Thus, the e-CuidateChemo program was successful in limiting the loss of strength of the musculature, which is essential for the development of daily activities, given its relation to the ability to move. These results are of vital importance, since chemotherapy induces wasting, weakness, and muscle fatigue [50], which could be reflected in the loss of strength or a minor improvement seen in the control group. Adams and collaborators [51] showed a reversion of sarcopenia with a moderate-resistance training program (between 60% and 70% of the maximum repetition) and a clinical improvement in the quality of life in patients with breast cancer during chemotherapy. Surprisingly, our program only has five basic resistance exercises of low intensity (ratings of 10-13 on the Borg scale), and these are sufficient to avoid the loss of strength and even improve it.

We did not find significant differences between groups in our handgrip strength or lumbar strength results. The constant use of these muscle in daily activities may have maintained this muscle in both groups. Furthermore, few exercises of these area (Table 1 and Textbox 1) were included in our training routine.

The analysis of our results related to anthropometric parameters and body composition showed a maintenance of these values, following the same trend in the control group. Kim and collaborators found a significant reduction of weight, body mass index, and body fat with a moderate-to-high intensity program based on walking during 12 weeks (5 consecutive days) [52]. However, our Web-based program was not tailored to improve these variables. Avoiding poor values related to these variable could be addressed in future studies, given their influence in lower physical conditions [53], chemotherapy toxicities [10,54], recurrence, or mortality [55,56] in patients with breast cancer.

The main strength of this work is that this is the first study, to our knowledge, that has tested the effectiveness of a low-intensity exercise program based on a Web-based system, to improve physical fitness, anthropometric parameters, and body composition in patients with breast cancer undergoing chemotherapy. Therefore, this study contributes to the current knowledge in this field. The e- CuidateChemo system could be an optimal alternative to support patients with breast cancer, preventing some of the barriers related to their participation in physical exercise programs and saving costs as compared to the high cost of face-to-face programs [15]. Nevertheless, some limitations should be noted. The use of a treadmill to develop the 6MWT is questionable due to a possible overestimation, despite the improvement in terms of percentages predicted in the walked distance. These results should be considered with prudence. Finally, a more extended study throughout the chemotherapy treatment could have produced different results. Thus, further studies are needed to improve the knowledge in this field and to examine whether the observed benefits continue after a long follow-up period.

In conclusion, this low-intensity Web-based exercise program is effective in reversing the detriment of the functional capacity and strength in patients with breast cancer undergoing chemotherapy. The e-CuidateChemo system could be an excellent option to limit the physical deterioration of patients with breast cancer undergoing chemotherapy, because it could prevent the known barriers to practice of physical exercise during chemotherapy [8].

## Appendix

Multimedia Appendix 1 CONSORT-EHEALTH checklist (V 1.6.1).

## Abbreviations

RCT: randomized controlled trial
